# Pathological Mechanisms of Bortezomib-Induced Peripheral Neuropathy

**DOI:** 10.3390/ijms22020888

**Published:** 2021-01-17

**Authors:** Shota Yamamoto, Nobuaki Egashira

**Affiliations:** 1 Department of Lipid Signaling, National Center for Global Health and Medicine, Tokyo 162-8655, Japan; syyamamoto@ri.ncgm.go.jp; 2 Department of Pharmacy, Kyushu University Hospital, Fukuoka 812-8582, Japan

**Keywords:** bortezomib, peripheral neuropathy, neurotoxicity, neuropathic pain, drug repositioning, drug repurposing

## Abstract

Bortezomib, a first-generation proteasome inhibitor widely used in chemotherapy for hematologic malignancy, has effective anti-cancer activity but often causes severe peripheral neuropathy. Although bortezomib-induced peripheral neuropathy (BIPN) is a dose-limiting toxicity, there are no recommended therapeutics for its prevention or treatment. One of the most critical problems is a lack of knowledge about pathological mechanisms of BIPN. Here, we summarize the known mechanisms of BIPN based on preclinical evidence, including morphological abnormalities, involvement of non-neuronal cells, oxidative stress, and alterations of transcriptional programs in both the peripheral and central nervous systems. Moreover, we describe the necessity of advancing studies that identify the potential efficacy of approved drugs on the basis of pathological mechanisms, as this is a convincing strategy for rapid translation to patients with cancer and BIPN.

## 1. Introduction

Bortezomib, a first-generation proteasome inhibitor, was approved by the US Food and Drug Administration (FDA) in 2003 for the treatment of multiple myeloma [1,2,3]. At present, bortezomib is often used for chemotherapy of multiple myeloma and mantle cell lymphoma. However, bortezomib-induced peripheral neuropathy (BIPN), characterized by numbness and painful paresthesia, remains one of the most troubling adverse reactions [4,5,6,7,8]. According to clinical practice guidelines released by the American Society of Clinical Oncology (ASCO) in 2014, there are no highly recommended therapies for the prevention or treatment of existing chemotherapy-induced peripheral neuropathy (CIPN), including BIPN [9]. Unfortunately, no drugs were added to the latest guidelines released in 2020 [10]. Therefore, novel therapies are highly desirable to prevent and manage CIPN symptoms.

Compared with several kinds of CIPN (such as those arising from platinum derivatives, taxanes, or vinca alkaloids), the pathological mechanisms of BIPN are poorly understood. Nevertheless, a variety of preclinical evidence has been generated over the last 5−10 years. While bortezomib does not penetrate into the central nervous system (CNS) due to the blood-brain barrier, it accumulates in the dorsal root ganglia (DRG) and causes neurotoxicity [11]. Several pathological aspects have been observed, such as morphological alteration of mitochondria and endoplasmic reticulum (ER), oxidative stress, sensitization of transient receptor potential ankyrin 1 (TRPA1), and neuroinflammation, which will be described later in this review. Furthermore, after accumulation in the DRG, bortezomib indirectly causes CNS dysfunction, including glial activation, disruption of glutamate homeostasis, and inflammation.

In this review, we describe cumulative preclinical evidence including up-to-date findings, and further summarize promising therapeutic targets to prevent and manage BIPN. In recent years, drug repositioning has been spotlighted as an efficient approach to develop novel analgesic drugs because approved drugs have already been examined for their safety and pharmacokinetics in humans [12]. Therefore, drug repositioning studies for BIPN have great potential for rapid translation to improve the quality-of-life of patients with cancer. Preclinical studies using approved drugs for BIPN therapy are also discussed.

## 2. Methodology

We searched PubMed using the terms “bortezomib neuropathy” and “bortezomib neurotoxicity”. Articles related to BIPN pathology and prevention and/or treatment of BIPN were manually selected on the basis of their originality and relevance to the scope of this review. We excluded articles written in any language other than English.

## 3. Characteristics of Bortezomib-Induced Peripheral Neuropathy

### 3.1. Pain Symptoms

Various rodent models have been established to study the mechanisms of BIPN. Although the common feature of these models is mechanical hypersensitivity (hyperalgesia and/or allodynia), these animals show no alteration of heat sensitivity [13,14,15,16,17,18,19]. Sensory fiber-specific nociception testing using a Neurometer (Neurotron Inc.) revealed that BIPN model mice injected with bortezomib developed sensitization of all three distinct fibers (unmyelinated C fibers and myelinated Aδ/Aβ fibers) [20]. With regard to cold sensitivity, however, there were discrepancies between models. Several studies demonstrated that administration of bortezomib induces cold hypersensitivity [13,15,16,17], while others observed no effect on cold sensitivity [18,19]. One possible explanation for this discrepancy is differences in the maximum blood concentration (C_max_) of bortezomib achieved in each study. Indeed, a dosage schedule of 0.2 mg/kg (rat) or 1.0 mg/kg (mouse) induced cold allodynia [13,15,16,17], but 0.15 mg/kg (rat) or 0.3 mg/kg (mouse) did not [18,19]. Importantly, a clinical trial revealed that subcutaneous injection of bortezomib causes peripheral neuropathy with lower incidence than intravenous injection [21]. Although its mechanisms remain unknown, the C_max_ of bortezomib is considered to be an important factor. Therefore, further analysis focusing on the interaction between the C_max_ of bortezomib and BIPN should be worthwhile.

### 3.2. Morphological Abnormalities

Histological analyses with light and electron microscopy have shown that bortezomib treatment induces morphological abnormalities in the peripheral nervous system (PNS). In the DRG, enlargement of satellite glial cells was observed, which might be associated with damage to their ER and mitochondria [22,23]. In contrast, it seems that no evident alteration occurs in the soma of sensory neurons [22,23].

Several studies have shown that bortezomib can elicit axonal degeneration in the sciatic nerve, as quantified by decreased circularity of axonal shapes [11,13,22]. In these degenerated axons, swollen and vacuolated mitochondria were observed [16]. Moreover, bortezomib was found to increase numbers of aberrant Schwann cells containing enlarged ER [23,24]. However, several studies reported that there were no obvious changes of myelin thickness in the sciatic nerve of bortezomib-treated animals compared with vehicle-treated groups.

Decreased density of intraepidermal nerve fibers (IENF) has also been observed in rats and mice treated with bortezomib [16,25,26]. However, alterations of IENF (increase/no change/decrease) in skin biopsies from patients remain controversial [27,28,29].

### 3.3. Involvement of Non-Neuronal Cells

As noted above, bortezomib administration induces morphological alterations of non-neuronal cells, such as satellite glial cells and Schwann cells. In addition, numbers of macrophages are reportedly increased in the DRG and sciatic nerve of bortezomib-treated animals; most of these infiltrating macrophages are inducible nitric oxide synthase (iNOS)-expressing inflammatory macrophages [30,31]. It is important to note that bortezomib not only affects the PNS, but also glial cells in the spinal cord, a part of the CNS. Specifically, a marked increase in the immunoreactivity of glial fibrillary acidic protein (GFAP), a well-known astrocytic marker, was observed in the superficial spinal dorsal horn [31,32,33]. These results indicate that bortezomib leads to increased numbers of astrocytes and/or their activation. In contrast, changes in microglial cell number and morphology were not observed [32].

## 4. Axonal Degeneration

### 4.1. Neurite Degeneration in Cell Culture Models

To elucidate mechanisms of and discover novel therapeutics for bortezomib-induced axonal degeneration, many studies have employed cell culture models that reflect in vivo observations. Most cell culture models utilized the PC12 cell line (rat pheochromocytoma 12) [13,34], SH-SY5Y cell line (human neuroblastoma) [35,36], or primary cultures of DRG neurons from rats or mice [26,37,38]. All culture models display reduced lengths of neurite outgrowth in response to bortezomib. However, a study establishing primary cultured DRG neurons from embryos of various strains (Sprague–Dawley rat, C57BL/6J mouse, DBA/2J mouse, BALB/cJ mouse, and C3H/HeJ mouse) demonstrated that the susceptibility to bortezomib-induced neurotoxicity differs among rodent strains [39]. Specifically, neurons from Sprague–Dawley rats and C57BL/6J mice are more sensitive than those from other strains [39].

Human induced pluripotent stem cell-derived sensory neurons have also been applied to evaluate the neurotoxicity of chemotherapies such as bortezomib, platinum agents, and paclitaxel [40]. This strategy is expected to be developed for pre-screening of BIPN in patients and examining neuroprotective drugs.

A study that independently manipulated cell bodies and axons of cultured DRG neurons using compartmentalized microfluidic chambers revealed that neurites remained intact following application of bortezomib to axons, but degenerated when cell bodies were exposed to bortezomib [26]. This study also demonstrated that bortezomib causes axonal degeneration via dual mechanisms. The major mechanism involves sterile alpha and toll/interleukin-1 receptor motif-containing 1 (SARM1), while the second is related to the caspase-3 mediated apoptosis pathway. Bortezomib exposure decreases nicotinamide mononucleotide adenylyltransferase 2 in axons, which triggers SARM1 activation followed by reduced axonal nicotinamide adenine dinucleotide (NAD^+^) levels, leading to axonal degeneration in cultured DRG neurons. Therefore, SARM1 and/or caspase inhibition could protect axonal degeneration caused by bortezomib [26].

### 4.2. Polymerization of Microtubules and Impairment of Axonal Transport

Although bortezomib is known as a proteasome inhibitor, increased tubulin polymerization has been observed in both in vivo and in vitro models of BIPN [11,36,37]. In addition, tubulin acetylation was observed in cell lines [36]. These alterations of intracellular tubulin dynamics may result in impairments of axonal transport. Indeed, bortezomib exposure decreased axonal transport of mitochondria and neurofilaments in culture models of DRG neurons [37,41]. In a mouse model, neurofilament proteins accumulated and the number of sensory neurons labeled with FluoroGold (a retrograde tracer) following injection into the tibial nerve was significantly decreased in the DRG of the bortezomib-treated group [41]. These studies indicate that bortezomib impairs axonal transport.

Notably, other proteasome inhibitors, such as lactacystin and MG-132, also increase polymerized tubulin [36,37]; thus, it is plausible that tubulin polymerization is the class effect of proteasome inhibitors. Therefore, further investigation is needed to elucidate the relationship among tubulin polymerization, axonal transport impairment, and BIPN.

## 5. Oxidative Stress

### 5.1. Impairment of Mitochondrial Function

Bortezomib causes morphological abnormalities within mitochondria of the PNS [16,23]. It has been demonstrated that the function of mitochondrial respiration and adenosine tri-phosphate (ATP) production is significantly reduced in bortezomib-treated animals [16,42]. Moreover, manganese superoxide dismutase, an important mitochondrial antioxidant enzyme, was excessively nitrated and exhibited decreased activity in nerves of bortezomib-treated rats. Thus, therapies that restore mitochondrial function could protect against bortezomib-induced mechanical hypersensitivity [16,42]. Furthermore, systemic administration of the reactive oxygen species (ROS) scavenger phenyl-N-tert-butylnitrone attenuated existing mechanical hyperalgesia induced by bortezomib [17].

### 5.2. Involvement of TRPA1 Channel

Several studies have implicated TRPA1 in bortezomib-induced neuropathic pain. Bortezomib could evoke hypersensitivity to allyl isothiocyanate, a TRPA1 agonist, following injection into the plantar of mice [15]. Genetic knockout of TRPA1 protected mice from developing mechanical and cold allodynia after bortezomib administration [15]. In addition, pharmacological inhibition of TRPA1 effectively attenuated neuropathic pain induced by bortezomib [43,44,45,46]. It has been reported that bortezomib increases protein expression of TRPA1, which is suppressed by inhibition of tumor necrosis factor alpha (TNF-α) or interleukin (IL)-6 signaling [45,46]. However, whether bortezomib alters TRPA1 protein expression remains controversial.

TRPA1 exhibits broad sensitivity for noxious cold, a variety of chemicals, hypoxia, low pH, and ROS [47,48]. Indeed, bortezomib causes oxidative stress, as observed by the accumulation of carbonylated proteins [49,50]. Moreover, it has been demonstrated that plasma levels of carboxy-methyl-lysine protein adducts are transiently increased one hour after bortezomib injection in mice [15]. Interestingly, early and short-term treatment with a TRPA1 antagonist or the oxidative scavenger α-lipoic acid completely prevented the development of bortezomib-induced sensory hypersensitivity. The treatment protocol with α-lipoic acid also abolished hypersensitivity to allyl isothiocyanate (a TRPA1 agonist) evoked by bortezomib [15]. This evidence indicates important roles of oxidative stress (such as that elicited by ROS)-triggered TRPA1 activation in bortezomib-induced sensory abnormalities.

## 6. Inflammatory Signaling in the PNS

### 6.1. Cytokines/Chemokines

In the rodent DRG, many types of inflammatory cytokines and chemokines are upregulated following bortezomib administration. Immunohistochemistry experiments demonstrated that increased TNF-α or CC chemokine ligand 2 (CCL2) signals co-localized with sensory neuron markers, while prokineticin-2 was increased in CD68-positive macrophages [25,31,51,52]. Moreover, pharmacological inhibition of cytokine/chemokine signals (such as TNF-α, IL-6, CCL2, and prokineticin-2) exerted protective effects against BIPN [31,46,51,52].

### 6.2. MAPKs

Mitogen-activated protein kinase (MAPK) signaling plays important roles in the development of BIPN. It has been shown that phosphorylation of c-Jun N-terminal kinase (JNK) and p38 MAPK are increased in DRG neurons [45,46,52,53]. In addition, pharmacological inhibition of these kinases was protective against bortezomib-induced sensory neuropathies [52,53]. Furthermore, inflammatory cytokine signals are often positioned upstream of MAPK activation. Indeed, excess phosphorylation of JNK by bortezomib treatment was suppressed in TNF-α receptor 1- or TNF-α receptor 2-knockout mice, which failed to develop bortezomib-induced neuropathic pain [52]. Inhibition of IL-6 signaling also effectively attenuated increases of JNK and p38 MAPK phosphorylation in bortezomib-treated rats [46].

### 6.3. Transcription Factors

Several studies have implicated dysregulation of transcription factors in the DRG in the development of BIPN. Following bortezomib administration, nuclear factor kappa-light-chain-enhancer of activated B cells (NF-κB) is increased in intranuclear fractions, indicating activation of the NF-κB signaling pathway [25]. Consistent with this finding, transgenic mice expressing a dominant negative form of IκBα (an endogenous inhibitor of NF-κB), which inhibited NF-κB translocation to the nucleus by preventing endogenous IκBα degradation, did not develop BIPN [54]. Moreover, bortezomib-induced axonal degeneration of the sciatic nerve was less severe in these transgenic mice, while decreased IENF density occurred at the same level observed in wild type mice [54].

Upregulation of activating transcription factor 3 (ATF3) is also reportedly involved in BIPN. In DRG neurons of BIPN rodents, ATF3-immunoreactivity was increased in the nucleus, whereas vehicle-treated animals rarely exhibited immunoreactivity [20,51]. The increase of ATF3 elicited by bortezomib enhanced the recruitment of c-Jun, a transcription factor known to form a heterodimer with ATF3 that binds the promoter region of *Ccl2*, ultimately resulting in upregulation of CCL2 and the development of neuropathic pain [51]. However, several other BIPN models did not cause upregulation of ATF3 expression in the DRG [13,16]. This discrepancy might arise from differences in dosages or schedules of bortezomib administration.

Signal transducer and activator of transcription-3 (STAT3) is also highly phosphorylated in DRG neurons following bortezomib injection [55]. Bortezomib causes an increase of NOD-like receptor family pyrin domain containing 3 (NLRP3) expression via enhancement of STAT3 binding to the promoter region of *Nlrp3*, as well as STAT3-dependent acetylation of histone H3/H4 in that region [55]. Indeed, both pharmacological or genetic inhibition of STAT3 and treatment of NLRP3 siRNA could prevent the development of bortezomib-induced mechanical allodynia in rats and mice [55].

## 7. Dysfunctions in the CNS

### 7.1. Glutamate Signaling

In addition to the PNS, bortezomib can lead to dysregulation in the CNS. As bortezomib cannot penetrate the blood-brain barrier [11], it likely causes CNS disruption in an indirect manner. Elevated concentrations of glutamate, the most major neurotransmitter, were observed in the cerebrospinal fluid of bortezomib-treated rats [56]. Moreover, bortezomib increased GFAP expression and altered astrocytic morphology in the spinal dorsal horn, while reducing the expression of glutamate/aspartate transporter (GLAST), an important extracellular glutamate uptake transporter expressed on astrocytes [57]. Thus, it is plausible that the elevation of glutamate concentration observed after bortezomib administration results from the downregulation of GLAST expression. Consistent with this notion, co-treatment with ceftriaxone (an activator of glutamate transporter activity) prevented bortezomib-induced mechanical allodynia and elevation of glutamate concentrations [57].

Disruption of spinal glutamate homeostasis can also affect the activity of wide dynamic range neurons in the spinal cord. Specifically, application of several noxious or innocuous mechanical stimuli to the paw enhanced responses and persistent after-discharges in wide dynamic range neurons of bortezomib-treated rats [19]. The persistent after-discharge elicited by bortezomib might result from reduced expression levels of GLAST.

In addition, electrophysiological experiments revealed bortezomib-induced alterations of the frequency of miniature excitatory postsynaptic currents (mEPSCs) in dorsal horn neurons of the spinal cord lamina II layer [33,58]. Cumulative evidence explains that the frequency and amplitude of mEPSCs indicates the probability of presynaptic neurotransmitter release and function of postsynaptic receptors, respectively. Increased frequency of mEPSCs, but not their amplitude [33,58], indicated increased presynaptic glutamate release occurred in bortezomib-treated animals.

### 7.2. Intracellular Signaling

MAPKs are highly phosphorylated in the spinal cord of BIPN rodent models. Following treatment with bortezomib (1 mg/kg, intraperitoneally, once weekly for 2 weeks), BALB/c mice exhibited increased phosphorylation of JNK and extracellular signal-regulated protein kinase (ERK) in the spinal cord [59]. In addition, inhibition of ERK phosphorylation could prevent the development of mechanical allodynia after bortezomib injection [59]. Astrocytic JNK is highly phosphorylated and involved in bortezomib-induced neuropathic pain, and could be attenuated by intrathecal injection of an inhibitor of TNF-α or IL-1 signaling [60]. In addition to ERK and JNK, phosphorylation of p38 MAPK is reportedly increased by bortezomib [53].

Several other intracellular kinases have been implicated in BIPN pathology. Bortezomib causes increased phosphorylation of protein kinase C (PKC) in the spinal cord, as well as its presynaptic membrane translocation [58,61], which triggers glutamate release and leads to bortezomib-induced sensory hypersensitivity [58]. In addition, phosphatidylinositol-3 kinase is activated in the spinal cord of BIPN rats [62]. Phosphorylation of phosphatidylinositol-3 kinase leads to further activation of mammalian target of rapamycin signaling. Thus, bortezomib might promote these signaling pathways to facilitate BIPN pathogenesis [62]. However, the upstream molecule that initiates pathological programs after bortezomib exposure is still unknown.

As described above, activation of STAT3 in the DRG is an important pathological mechanism of BIPN. Bortezomib has also been shown to increase STAT3 phosphorylation in dorsal horn neurons of the spinal cord, which is induced following downregulation of sirtuin 1 [63]. Activated STAT3 is recruited to the promoter region of NACHT leucine-rich-repeat protein 1 (an inflammasome family molecule) and increases histone acetylation, resulting in bortezomib-induced mechanical allodynia. Treatment with the sirtuin 1 activator resveratrol could ameliorate phosphorylation of STAT3 and increase expression of NACHT leucine-rich-repeat protein 1, which suppressed the development of mechanical allodynia following bortezomib administration [63].

### 7.3. Diffusible Factors

A number of cytokines and chemokines in the spinal cord are affected by bortezomib. Specifically, expression of TNF-α and CCL21 were increased in dorsal horn neurons, while increased IL-1β and prokineticin-2 expression were observed in astrocytes [31,53,60,64]. In contrast, bortezomib decreased anti-inflammatory cytokines IL-4 and IL-10 in the spinal cord [31,33]. Inhibition of these inflammatory cytokine signals in the spinal cord could prevent the development of neuropathic pain after bortezomib treatment [31,60,64].

Recently, contributions of dysregulated sphingolipid metabolism to BIPN have been reported [33]. Sphingolipid metabolite sphingosine-1-phosphate (S1P), a well-known bioactive lipid mediator, exerts its biological roles through S1P receptors (S1PRs) [65,66]. Bortezomib administration activates the *de novo* sphingolipid synthesis pathway in the spinal dorsal horn, resulting in increases of S1P, ceramide, and several related metabolites [33]. Moreover, co-treatment with an inhibitor of the rate-limiting enzyme serine palmitoyltransferase or S1PR1 antagonists ameliorated the development of bortezomib-induced mechanical hypersensitivity. Notably, antagonism of S1PR1 prevented increases of GFAP immunoreactivity, astrocytic morphological alterations, upregulation of presynaptic glutamate release, and changes of cytokine expression. Furthermore, astrocyte specific S1PR1 knockout could mimic these protective effects against BIPN [33], indicating important roles of astrocytic S1PR1 in the pathogenesis of BIPN.

## 8. Proteasome Inhibition

The involvement of proteasome inhibition in BIPN pathology remains an unsolved and important question in the field, although several studies have addressed this challenge. Bortezomib reportedly inhibits proteasome activity in the blood, sciatic nerve, and DRG, but not in the brain of rats after intravenous injection [11]. In addition, bortezomib-induced proteasome inhibition leads to increased expression of T-type calcium channels (Ca_v_3.2) both in vivo and in vitro, which causes mechanical allodynia [67]. However, exposure of a DRG neuron-derived cell line to another proteasome inhibitor, MG-132, could mimic this increase [67].

Carfilzomib, a second-generation proteasome inhibitor, is known to induce peripheral neuropathy with low frequency compared with bortezomib. A study using cellular models demonstrated that bortezomib exposure causes reduced neurite outgrowth, but carfilzomib does not, despite equivalent levels of proteasome inhibition [35]. These results suggest the involvement of another factor in the development of peripheral neuropathy.

Off-target screening identified the serine protease cathepsin G as a non-proteasomal target of bortezomib, and activity of cathepsin G in rat splenocytes was decreased after injection of bortezomib, but not carfilzomib. Moreover, cathepsin G activity in blood of bortezomib-treated patients was decreased [35]. Proteomic approaches revealed that treatment of neuronal cells with bortezomib induced more severe protein oxidation than carfilzomib [49,50]. Although these findings provide some clues, further investigation is necessary to understand the non-proteasomal mechanisms of BIPN.

## 9. Therapeutics Approaches of BIPN

### 9.1. Analgesic Drugs and Adjuvants

Several analgesic drugs and adjuvants have validated efficacy against bortezomib-induced neuropathic pain. Among them, pregabalin is the most frequently used analgesic drug in clinical practice. In fact, pregabalin attenuated existing mechanical allodynia in BIPN rats [13]. In addition, it was demonstrated that bortezomib-induced mechanical allodynia could be reversed by systemic administration of gabapentin, tramadol, duloxetine, or mexiletine [13,18]. In contrast, diclofenac or amitriptyline did not exert anti-allodynic effects in a BIPN model [13]. Goshajinkigan, an herbal medicine widely used for treatment of numbness and limb pain, also reportedly reduces mechanical allodynia in bortezomib-treated rats via a kappa opioid receptor-dependent mechanism [68]. Unfortunately, except for duloxetine, none of these drugs are recommended or have been examined for efficacy against CIPN in clinical trials [9,10]. According to ASCO guidelines, only duloxetine is moderately recommended for the treatment of existing CIPN [9,10].

### 9.2. Antioxidants and TRPA1 Inhibitors

Antioxidant therapy has also been examined in rodent models. Co-treatment with acetyl-L-carnitine prevented the development of sensory neuropathy and mitochondrial dysfunction following bortezomib administration [16]. Moreover, oxidative scavengers could both prevent and reverse bortezomib-induced allodynia in rodents [15,17]. However, it was reported that the antioxidant α-lipoic acid can affect the anti-tumor activity of bortezomib in melanoma cells [69]. Thus, it is difficult to recommend strategies targeting pathological events related to the mechanisms of bortezomib-induced anti-cancer effects.

Oxidative stress–induced TRPA1 activation should also be a therapeutic target against BIPN. Unlike targeting of oxidative stress itself, inhibition of TRPA1 channels might be a promising strategy. TRPA1 is involved in both BIPN [15,43,44] and CIPN induced by drugs such as oxaliplatin or paclitaxel [70,71]. TRPA1 has been focused on as a novel therapeutic target for pathological pain states and respiratory disorders [72,73]. Thus, obtaining further clinical evidence for novel analgesics targeting TRPA1 channels is worthwhile.

### 9.3. Targeting Glial Dysfunction

Several studies have revealed that spinal cord astrocytic activation is responsible for the development of BIPN, as described above. Bortezomib-induced sensory neuropathy and activation of astrocytes was prevented by co-treatment with the glial inhibitor minocycline [32]. Disruption of glutamate homeostasis also seems to be a promising target. Indeed, ceftriaxone could suppress BIPN symptoms in rats [57].

Bortezomib dysregulates sphingolipid metabolism in the spinal cord; specifically, enhanced S1P-S1PR1 signaling in astrocytes leads to spinal cord neuroinflammation and BIPN [33]. The FDA-approved drug fingolimod can prevent and, importantly, reverse bortezomib-induced neuropathic pain [33]. In addition, fingolimod exerts protective effects to other neurotoxic chemotherapy-induced allodynia [74]. As fingolimod is now approved for multiple sclerosis, the results of these studies suggest that drug repositioning clinical trials should be advanced. However, given that S1PR1 antagonism could prevent BIPN only in male rodents [75], potential sex-based differences in the protective effect of fingolimod should be considered.

### 9.4. Others

Drug repositioning is one of the most promising strategies to rapidly translate outcomes to patients. Recently, researchers have embraced drug-repositioning studies, the results of which have demonstrated that several types of FDA-approved drugs have protective effects against BIPN.

T-type calcium channels are reportedly increased in the DRG following bortezomib injection [67]. Consistent with this evidence, the T-type calcium channel inhibitor ethosuximide exerts an anti-allodynic effect against existing BIPN [17]. Clinical trials examining the efficacy of ethosuximide against neuropathic pain (NCT04431778) and visceral pain (NCT02973542) are ongoing.

Metformin, which is well-known to exert pleiotropic effects, prevented paclitaxel- and cisplatin-induced CIPN in mice [76]. With regard to BIPN, it has been demonstrated that metformin prevents its development by hypoxia-inducible factor 1α-related mechanisms [77]. Hypoxia-inducible factor 1α is responsible for oxaliplatin-induced TRPA1 sensitization and cold allodynia [78], and a clinical trial reported that metformin reduced the severity of oxaliplatin-induced CIPN [79]. Thus, drug repositioning of metformin as an anti-CIPN drug is expected in the future.

Targeting of intracellular kinase activation has been examined to treat BIPN. Tamoxifen and trametinib, which are FDA approved for cancer therapy, were shown to suppress bortezomib-induced sensory neuropathy by inhibiting phosphorylation of PKC and ERK, respectively [59,61]. Given that chemotherapeutic regimens are often composed of multiple anticancer drugs, the possibility exists that standard regimens plus PKC or ERK inhibitors could successfully attenuate CIPN.

## 10. Future Perspectives

**Figure 1:**
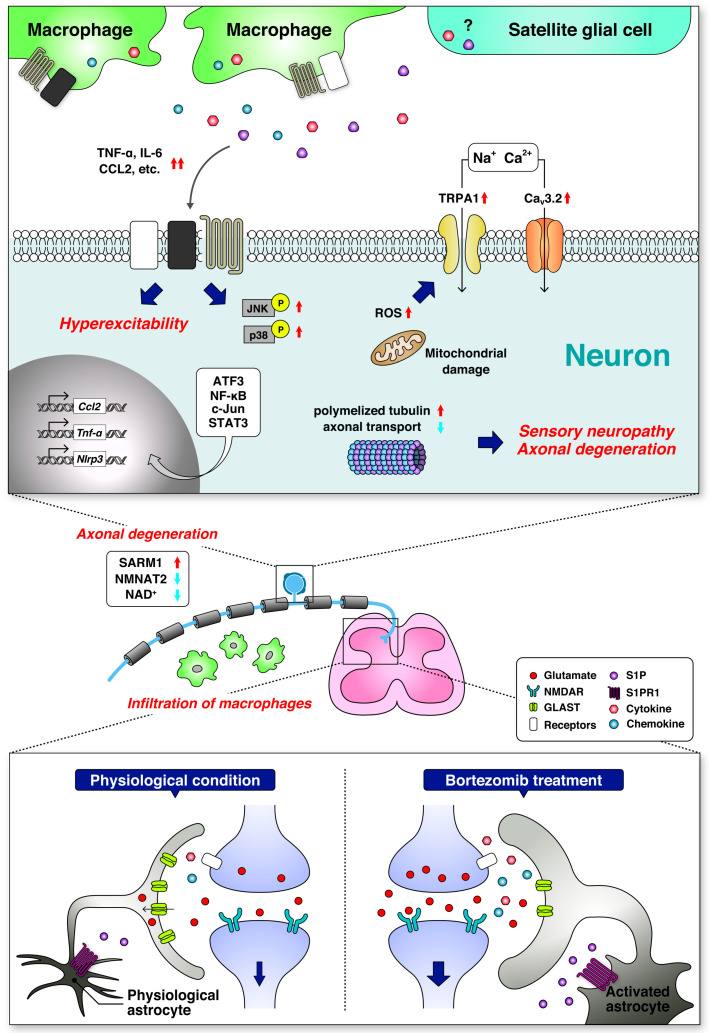
Mechanisms of bortezomib-induced peripheral neuropathy (BIPN). Schematic illustrations show the proposed principal pathways dysregulated by bortezomib in the dorsal root ganglia (DRG) and spinal dorsal horn involving BIPN. In the DRG, bortezomib increases polymerized tubulin and decreases axonal transport. In addition, NAD^+^ levels are decreased in axons, leading to axonal degeneration. Bortezomib impairs mitochondrial function and increases ROS production. Excessive ROS sensitizes TRPA1, resulting in sensory hypersensitivities. Bortezomib also enhances transcriptional programs, which causes inflammatory events and neuronal hyperexcitability. In the dorsal horn, bortezomib dysregulates sphingolipid metabolism, leading to astrocytic activation, neuroinflammation, and disruption of glutamate homeostasis. Finally, these abnormalities result in bortezomib-induced sensory neuropathy. Red up arrows indicate an increase and/or activation, while blue down arrows indicate a decrease and/or inhibition. Abbreviations: activating transcription factor 3 (ATF3); voltage-gated calcium channel 3.2 (Ca_v_3.2); CC chemokine ligand 2 (CCL2); glutamate/aspartate transporter (GLAST); interleukin-6 (IL-6); c-Jun N-terminal kinase (JNK); nicotinamide adenine dinucleotide (NAD^+^); nuclear factor kappa-light-chain-enhancer of activated B cells (NF-κB); NOD-like receptor family pyrin domain containing 3 (NLRP3); N-methyl-d-aspartate receptor (NMDAR); nicotinamide mononucleotide adenylyltransferase 2 (NMNAT2); reactive oxygen species (ROS); sterile alpha and toll/interleukin-1 receptor motif-containing 1 (SARM1); sphingosine-1-phosphate (S1P); sphingosine-1-phosphate receptor 1 (S1PR1); signal transducer and activator of transcription-3 (STAT3); tumor necrosis factor alpha (TNF-α); transient receptor potential ankyrin 1 (TRPA1).

In this review, we summarized preclinical evidence including the characteristics, pathological mechanisms, and therapeutic possibilities of BIPN (Figure 1). Unfortunately, few clinical trials aimed to overcome BIPN based on preclinical evidence, although other CIPN-related clinical studies are now underway [80,81]. Thus, we look forward to translating the preclinical evidence reviewed here into clinical practice.

Although each chemotherapeutic drug induces peripheral neuropathy by distinct mechanisms, there are nevertheless common aspects among several CIPN. For example, the involvement of S1P and S1PR1 signaling is a common mechanism among oxaliplatin-, paclitaxel-, and bortezomib-induced CIPN [33,74,75]. At present, clinical trials (NCT03941743 and NCT03943498) are ongoing to examine whether fingolimod can reduce paclitaxel-induced peripheral neuropathy. In addition, axonal degeneration of the sciatic nerve is a common feature of CIPN. NAD^+^ reduction is reportedly involved in paclitaxel-, bortezomib-, and vincristine-induced CIPN [26,82], and pharmacological strategies to increase NAD^+^ levels can prevent or reverse CIPN-related pain [26,82,83]. Indeed, nicotinamide riboside, a recently discovered vitamin precursor of NAD^+^, is currently being examined for its efficacy against CIPN (NCT04112641). Therefore, it is expected that the efficacy of fingolimod or nicotinamide riboside against BIPN will also be examined in the near future.

Over the last 5−10 years, preclinical data have identified the transporters responsible for the development of CIPN, such as oxaliplatin and paclitaxel [84,85,86,87]. However, it remains unclear which transporter is responsible for bortezomib accumulation in the DRG [88]. Additionally, it is also unknown which cell type (sensory neurons, macrophages, satellite glial cells, or others) uptakes bortezomib, leading to the development of BIPN. If we could limit inhibition of bortezomib uptake to the DRG (or other nervous system) alone, this would be a promising strategy to prevent BIPN, although the oncological safety must be considered. Therefore, identification of the entry pathways of bortezomib into the nervous system and cancer cells should be addressed.

A biomarker reflecting the severity of pain is strongly desired for both diagnostics in clinical practice and validation of the efficacy of novel analgesic drugs in clinical trials. Recently, lipidomics analysis of serum from 59 patients identified certain lipid species as biomarker candidates of BIPN symptoms [89]. Although further validation is necessary, several species of phosphatidylcholines, ceramides, neutral lipids, and oxidative fatty acids seem to be related to the severity of BIPN [89]. These approaches exploring symptom-reflecting biomarkers will not only facilitate objective visualization of the severities of symptoms, such as pain, but also improve our understanding of the pathological mechanisms of BIPN.

We sincerely hope that the preclinical evidence reviewed here will be evaluated in clinical trials and result in improved quality-of-life for patients with cancer.

## Abbreviations

ASCO	American Society of Clinical Oncology
ATF3	Activating transcription factor 3
BIPN	Bortezomib-induced peripheral neuropathy
CCL	CC chemokine ligand
CIPN	Chemotherapy-induced peripheral neuropathy
C_max_	Maximum blood concentration
CNS	Central nervous system
DRG	Dorsal root ganglia
ER	Endoplasmic reticulum
ERK	Extracellular signal-regulated protein kinase
FDA	the US Food and Drug Administration
GFAP	Glial fibrillary acidic protein
GLAST	Glutamate/aspartate transporter
IENF	Intraepidermal nerve fiber
IL	Interleukin
JNK	c-Jun N-terminal kinase
MAPK	Mitogen-activated protein kinase
mEPSCs	Miniature excitatory postsynaptic currents
NAD^+^	Nicotinamide adenine dinucleotide
NF-κB	Nuclear factor kappa-light-chain-enhancer of activated B cells
NLRP3	NOD-like receptor family pyrin domain containing 3
PNS	Peripheral nervous system
PKC	Protein kinase C
ROS	Reactive oxygen species
SARM1	Sterile alpha and toll/interleukin-1 receptor motif-containing 1
S1P	Sphingosine-1-phosphate
S1PR	Sphingosine-1-phosphate receptor
STAT3	Signal transducer and activator of transcription-3
TNF-α	Tumor necrosis factor alpha
TRPA1	Transient receptor potential ankyrin 1
